# Alzheimer’s Disease Pathology and Assistive Nanotheranostic Approaches for Its Therapeutic Interventions

**DOI:** 10.3390/ijms25179690

**Published:** 2024-09-07

**Authors:** Anuvab Dey, Subhrojyoti Ghosh, Ramya Lakshmi Rajendran, Tiyasa Bhuniya, Purbasha Das, Bidyabati Bhattacharjee, Sagnik Das, Atharva Anand Mahajan, Anushka Samant, Anand Krishnan, Byeong-Cheol Ahn, Prakash Gangadaran

**Affiliations:** 1 Department of Biosciences and Bioengineering, Indian Institute of Technology Guwahati, North Guwahati 781039, Assam, India; anuvab2000dey@gmail.com; 2 Department of Biotechnology, Indian Institute of Technology Madras, Chennai 600036, Tamil Nadu, India; subhrojyotighosh8@gmail.com; 3 Department of Nuclear Medicine, School of Medicine, Kyungpook National University, Daegu 41944, Republic of Korea; ramyag@knu.ac.kr; 4 Department of Biotechnology, National Institute of Technology Durgapur, Durgapur 713209, West Bengal, India; tiyasa28082000@gmail.com; 5 Department of Life Sciences, Presidency University, Kolkata 700073, West Bengal, India; purbasha.mantai@gmail.com; 6 Department of Life Sciences, Jain (Deemed-to-be) University, Bangalore 560078, Karnataka, India; bidyabatibhattacharjee@gmail.com; 7 Department of Microbiology, St Xavier’s College (Autonomous), Kolkata 700016, West Bengal, India; sagnikd525@gmail.com; 8 Advance Centre for Treatment, Research and Education in Cancer (ACTREC), Navi Mumbai 410210, Maharashtra, India; 9 Department of Biotechnology and Medical Engineering, National Institute of Technology, Rourkela, Rourkela 769008, Orissa, India; anushkas2709@gmail.com; 10 Department of Chemical Pathology, School of Pathology, Office of the Dean, Faculty of Health Sciences, University of the Free State, Bloemfontein 9300, South Africa; krishnana1@ufs.ac.za; 11 Department of Nuclear Medicine, Kyungpook National University Hospital, Daegu 41944, Republic of Korea; 12 BK21 FOUR KNU Convergence Educational Program of Biomedical Sciences for Creative Future Talents, Department of Biomedical Sciences, School of Medicine, Kyungpook National University, Daegu 41944, Republic of Korea

**Keywords:** Alzheimer’s disease, neurodegeneration, nanotheranostic, nanomedicine, personalized therapy

## Abstract

Alzheimer’s disease (AD) still prevails and continues to increase indiscriminately throughout the 21st century, and is thus responsible for the depreciating quality of health and associated sectors. AD is a progressive neurodegenerative disorder marked by a significant amassment of beta-amyloid plaques and neurofibrillary tangles near the hippocampus, leading to the consequent loss of cognitive abilities. Conventionally, amyloid and tau hypotheses have been established as the most prominent in providing detailed insight into the disease pathogenesis and revealing the associative biomarkers intricately involved in AD progression. Nanotheranostic deliberates rational thought toward designing efficacious nanosystems and strategic endeavors for AD diagnosis and therapeutic implications. The exceeding advancements in this field enable the scientific community to envisage and conceptualize pharmacokinetic monitoring of the drug, sustained and targeted drug delivery responses, fabrication of anti-amyloid therapeutics, and enhanced accumulation of the targeted drug across the blood–brain barrier (BBB), thus giving an optimistic approach towards personalized and precision medicine. Current methods idealized on the design and bioengineering of an array of nanoparticulate systems offer higher affinity towards neurocapillary endothelial cells and the BBB. They have recently attracted intriguing attention to the early diagnostic and therapeutic measures taken to manage the progression of the disease. In this article, we tend to furnish a comprehensive outlook, the detailed mechanism of conventional AD pathogenesis, and new findings. We also summarize the shortcomings in diagnostic, prognostic, and therapeutic approaches undertaken to alleviate AD, thus providing a unique window towards nanotheranostic advancements without disregarding potential drawbacks, side effects, and safety concerns.

## 1. Introduction

Alzheimer’s disease (AD) is a chronic progressive neurological ailment for which there are currently few drug alternatives or other forms of treatment that can prevent or slow the progression of AD [1]. Currently, it is the most prevalent form of dementia in older adults. The characteristic hallmarks of this disease include significant loss of memory and sporadic memory impairment in the early phases of the illness [2,3]. There are concerns regarding the disorders linked to AD and how they progress exactly, considering the research suggesting that plaques started to form before the emergence of clinical signs by 20 years [4]. Age, gender, weight, physical activity, toxins, brain injury, and common hereditary variables (such as genetic abnormalities in the PS1, PS2, and APP proteins in familial AD) have all played a role in the beginning and development of AD pathogenesis in recent years. AD causes dementia in 36 million adults over 65 worldwide. Their numbers are anticipated to double by 2030 to 66 million and by 115 million by 2050 [5,6]. According to extensive studies, numerous cellular mechanisms, including amyloid beta (Aβ) accumulation, mitochondrial structural and functional alterations, the hyper-phosphorylation of tau and intracellular neurofibrillary tangle (NFT) formation, neuronal loss, and inflammatory responses have been implicated in the etiology of AD [7]. There is a shred of strong evidence claiming the contribution of oxidative stress in damaging cells and DNA, leading to the formation of lesions and plaques. Other dysregulations implicated in AD include mitochondrial dysfunction, protein misfolding, and inflammation. Each of these plays a role in the pathogenesis of the disease [8]. Major areas of current research include mitochondrial abnormalities, phosphorylated tau, and amyloid beta-induced synaptic damage. In the early stages of illness progression, mitochondrial oxidative damage and synaptic degeneration are observed [9]. Although numerous studies have substantially increased our understanding of AD, more research is still needed to elucidate the precise mechanism underlying its intricate etiology. To maintain Ca^2+^ homeostasis, mitochondria provide buffering machinery, which aids in the excitation of neuronal cells. Additionally, it shields neuronal cells throughout their lengthy neuronal lifetimes from several stresses. As a result, mitochondria are essential for intracellular communication, stress response, oxidative phosphorylation (OXPHOS), and the production of reactive oxygen species (ROS) [9]. Due to their high level of dynamic activity, mitochondria also influence the morphology of neuronal cells and where they are located inside these cells. 

Apart from the complex pathology and intricate molecular mechanisms underlying AD progression, prompt diagnosis remains shrouded with uncertainties and ineffective biomarker prediction. The main techniques for accurately diagnosing AD, which is still a difficult task, include neuroimaging techniques like magnetic resonance imaging (MRI) and amyloid positron emission tomography (PET) imaging, or cerebrospinal fluid (CSF) examination. While effective, these techniques are expensive, time-consuming, and beyond the means of most individuals. In this situation, nano biosensors are proposed as promising replacements for rapid, affordable, and simple AD diagnosis. A positive biomarker pattern for AD can be detected in other brain illnesses when AD pathology is present as a comorbidity. One of AD’s more advanced diagnostic and imaging methods is the sensitive early phase detection of AD biomarkers like Aβ and tau using nanoparticles (NP). The blood–brain barrier (BBB) controls the flow of biomolecules between blood arteries and brain cells in both directions. Developing efficient drug delivery systems for the brain is significantly hampered by the BBB. One of the major challenges to developing potent drugs for the prevention and treatment of AD is the selective nature of the BBB, which prevents many medicines for the central nervous system (CNS) from entering the brain. This clarifies why just 5% of medicines can enter the brain via passive diffusion [10]. Another significant barrier to the systemic treatment of brain illnesses is the need to provide CNS medications at large doses to achieve appropriate therapeutic efficacy, resulting in considerable peripheral adverse effects [11]. 

**Figure 1:**
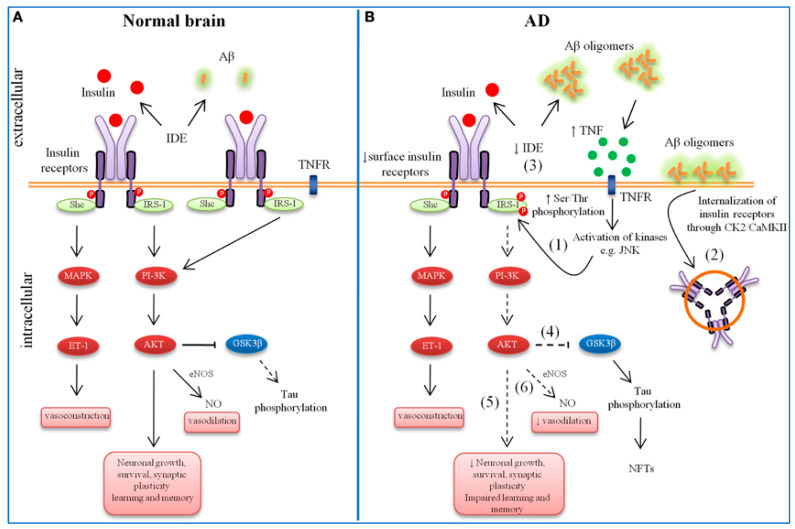
In a healthy brain (**A**), insulin binding to its receptor activates IRS-1 and PI3K, supporting neuronal health, growth, and cognitive functions. This process also balances blood vessel dilation and constriction to meet metabolic needs. In AD (**B**), Aβ oligomers disrupt this system by increasing TNF-α and activating stress kinases like JNK, which negatively affects IRS-1 (1). These oligomers also displace insulin receptors (IRs) from the cell surface by the actions of CK2 and CaMKII, relocating them away from areas where they are needed (2). This leads to insulin resistance, decreasing Aβ-degrading enzyme (IDE) levels (3), thus reducing Aβ clearance. The impaired insulin signaling escalates GSK-3β activity (4), promoting abnormal tau phosphorylation and damaging neuronal functions and cognitive abilities (5). Furthermore, this dysfunction disrupts vascular regulation (6), reducing nitric oxide (NO) production, decreasing cerebral blood flow, and increasing inflammation and oxidative stress (reprinted with permission from ref [17] with CC BY license Copyright© 2015 Bedse, Di Domenico, Serviddio and Cassano). CaMKII—Calcium/calmodulin-dependent protein kinase II; CK2—Casein kinase 2; eNOS—Endothelial nitric oxide synthase; ET—Endothelin; GSK-3β—Glycogen synthase kinase-3 beta; IDE—Insulin-degrading enzyme; IRS-1—Insulin receptor substrate 1; JNK—c-Jun N-terminal kinase; NO—Nitric oxide; PI3K—Phosphoinositide 3-kinase; TNF-α—Tumor necrosis factor-alpha; and TNFR—Tumor necrosis factor receptor.

To circumvent the BBB’s restrictions, numerous colloidal delivery systems have been developed during the past 20 years that use the benefits of particle size reduction. These systems comprise solid lipid NPs, cubosomes, metal NP-based carriers, polymeric NPs, liposomes, metal NPs, and emulsions [12]. Creating these NPs for drug delivery is a diverse field that provides fresh perspectives and opens up possibilities for working with materials with at least one dimension between 1 and 150 nm in size [9]. NPs are superior to traditional drug delivery molecules in a variety of ways. As a result of their small size and high surface-to-volume ratio, they can better interact with biomolecules. They can be designed in several sizes and shapes to alter their passage through biological barriers, including spherical, cubic, and rod-like forms [13]. NPs can attach to various useful ligands to gain new physiological, therapeutic, or diagnostic properties via adsorption, entrapment, or covalent connections. It is also important to consider whether these particles could lead to an innate immune response [14]. Numerous sectors, including medicine, pharmacy, chemistry, and biological detection, have greatly benefited from advances in nanomedicine [15]. For the prevention and treatment of AD, functionalized nanomaterials, which have been used extensively to suppress Aβ protein aggregation, hold immense promise [16] (Figure 1).

Various ligands with the appropriate ligand density and receptor affinity are attached to the surface of NPs, and their physicochemical properties are altered to facilitate drug administration. The use of engineered nanomaterials is gaining popularity due to their tiny size and relatively high surface-to-volume ratios, which increase the chances of nanoparticle exposure [18]. Additionally, NPs can enter the brain. It is believed that NPs that adhere to the nasal mucosa of the olfactory bulb travel via the olfactory nerve to the olfactory bulb, where they can circulate to the brain and potentially affect the well-being and activities of the brain [19]. NPs have been the subject of intense research as drug delivery systems in recent years because they can transport medications precisely to specific sites, potentially limiting any negative side effects [20]. NPs have also been investigated for the treatment of AD, even though they can enhance medication targeting and increase drug availability in the CNS [21]. Nanotechnology presents a fresh approach to creating alternative drug delivery solutions for all stages of AD. Many NPs, including titanium dioxide, silica dioxide, silver, and zinc oxide, have been used in studies to treat neurological diseases. In the past ten years, there has been much interest in combining oxidant NPs with bioactive antioxidants like selenium (Se) and flavonoids, polyphenols, and other phytochemicals in AD [16]. These NPs have been demonstrated to have pro-inflammatory effects in in vitro and in vivo immune response studies [22]. AD and other CNS illnesses commonly involve inflammation [23]. Due to this paradigm, immunological responses to NPs are highly diverse. Still, they can also frequently be tailored to prevent unwanted immune responses and, in certain circumstances, even elicit favorable ones [24]. Despite the potential for some forms of NPs to accelerate AD development, a recent study reveals that specific sizes, shapes, and types of sulfur NPs (SNPs) can moderate AD pathogenesis [25]. The effects of three different types of brain-targeting SNPs (RVG, Met, SNPs) with new morphologies—volute-like, tadpole-like, and sphere-like—on BBB penetration and overall neurotoxicity have been investigated. In contrast to larger NPs that may enhance Aβ peptide aggregation and aggravate AD pathogenesis, smaller NPs, like sphere-shaped SNPs, may inhibit Aβ peptide aggregation (61.6%) and increase cell survival (92.4%) [26]. 

Despite having unique physical and chemical features that explain their wide variety of applications for the CNS, NPs can have neurotoxic effects due to cell necrosis, the production of free radicals, immunological responses, and neuroinflammation [27]. Reactive oxygen species (ROS), the main mechanism causing toxicity, may be produced when NPs are exposed, according to in vitro and in vivo studies [28]. Thus, this review article emphasizes the mechanistic principle underlying AD pathogenesis’s unknown facets and the role of nanoscience in the prompt diagnosis, prognosis, and development of novel therapeutic cargos against pathogenic biomarkers.

## 2. Role of Aβ in AD

Toxic changes occurring in the brain at the preliminary stages of AD include aberrant protein buildups that result in amyloid plaques and tau tangles. In individuals with sporadic AD, brain Aβ is comparatively high [29]. The primary component of cerebral parenchyma and vascular amyloid, Aβ, is neurotoxic and contributes to cerebrovascular lesions. How exactly Aβ builds up in the brain and causes cell disease remains unclear. AD development may be significantly influenced by the Aβ, according to evidence from pathologic, genetic, biologic, and biomarkers [30,31,32,33]. While amyloid plaques are a neuropathological indicator of AD, Aβ is a standard peptide produced throughout life. Nevertheless, synaptic activity, the most distinctive and typical aspect of the nervous system, stimulates Aβ production and secretion. The small Aβ peptide, which can be up to 42 or 43 amino acids long, is therefore not necessarily harmful and may perhaps serve a physiological function, in contrast to amyloid plaques, which are made up of numerous highly aggregated Aβ fibrils [29,34]. In AD brains, this naturally occurring protein assembles improperly to form plaques that amass between neurons and impede cell activity. The primary targets for treating AD are tau and Aβ proteins. As AD progresses, Aβ proteins generate oligomers and extracellular plaques that can transfer from one cell to another, potentially spreading the disease [35]. Soluble Aβ oligomers contribute to neurotoxicity by interacting with the lipid membrane, weakening its integrity, and interfering with receptor function [36]. A significant amount of genetic, neuropathological, and experimental evidence substantiates the amyloid cascade hypothesis, which associates Aβ aggregation with the cognitive manifestations of AD. As per the amyloid cascade hypothesis, the accumulation of amyloid peptide in the brain tissue is a crucial stage in the advancement of AD [37,38]. According to biological studies, when the APP, PS1, and PS2 proteins are altered, more disease-associated Aβ42 and other Aβ forms more prone to aggregation are created [39]. To conclude, investigations into biomarkers present in cerebrospinal fluid (CSF) indicate a reduction in Aβ42 peptides, which are associated with the disease, 10 to 20 years before the onset of AD symptoms [40,41]. Thus, Aβ has significantly contributed towards the initiation, pathogenesis, and progression of AD invariably, emerging as a prominent biomarker for disease detection and subsequent therapeutic alleviation.

## 3. Role of Tau and Dysregulated Phosphorylation in AD

Tau protein, a microtubule-associated protein (MAP) family member, influences axonal transport and growth, neuronal polarization, and, consequently, normal brain and neural activity [42,43]. Pathologically, insoluble tau aggregates accumulate in neurons, extracellular space, and other brain cells, such as oligodendrocytes and astrocytes [44,45]. Neurodegenerative diseases, such as AD, are distinguished by the formation of paired helical filaments (PHF) and NFT, which are produced by abnormally mutated and truncated tau proteins [46]. As per Hanseeuw et al., tau NFTs are frequently associated with clinical symptoms and neuronal loss. While Aβ may initiate a chain of events, tau impairment is more likely to be the effector molecule of neurodegeneration. Tau is essential in various physiological processes, including axonal transport, neurogenesis, motor function, learning, memory, neuronal excitability, glucose metabolism, iron homeostasis, and DNA protection [47]. It should be noted that tau pathology can be found in tauopathies, a group of neurodegenerative diseases distinct from AD. However, it is primarily expressed in the central and peripheral nervous systems, with the highest concentration in nerve cell axons. AD is characterized by tau buildup, but this pathology can also be present in other conditions [34]. Morris et al. reported that tau undergoes various post-translational modifications in the brain, including phosphorylation, acetylation, methylation, glycation, isomerization, O-Glc-N-Acetylation, nitration, SUMOylation, ubiquitination, and truncation. However, it is still unclear what function each of these modifications serves for tau. Phosphorylation is the most researched tau post-translational modification. Growing evidence shows phosphorylated tau (P-tau) prevents mitochondria and other subcellular organelles from traveling down axons in AD neurons [48]. Tau aggregates build up in the entorhinal cortex and hippocampus first before migrating to other regions in a highly predictable manner in AD. According to Braak’s staging of AD, because of how it often manifests in nerve cells, inclusions first appear in subcortical regions, trans-entorhinal cortex, and entorhinal cortex (stages I and II). Stages III and IV of the neocortex and the hippocampal formation follow, and then most of the neocortex (stages V and VI). People in stages I and II are asymptomatic, some in stages III and IV have symptoms of memory loss, and people in stages V and VI experience AD [30,49,50].

Two neuropathological indicators of AD include amyloid plaques, predominantly made of aggregated Aβ, and NFTs, made of tau, a protein connected to microtubules. The APP is altered in familial types of AD. While P-tau and Aβ are thought to contribute to the onset of the disease [51], an increasing body of research suggests that P-tau influences how subcellular organelles, such as mitochondria, lysosomes, vesicles, and proteins, are transported from the cell soma to nerve terminals through axons, which may have an impact on the pathogenesis of AD [26,50]. P-tau, linked to synaptic dysfunction and AD, has generated much interest as a potential therapeutic target.

Numerous studies have demonstrated that abnormally high quantities of either mutant or normal tau tend to be hyperphosphorylated in neurons, resulting in oxidative stress, mitochondrial dysfunction, synaptic depletion, and neuronal death. This discovery was made using brain tissue from transgenic mouse tau, APP/PS1, and 3XAD49 models. There have been reports of hyperphosphorylated tau, oxidative damage, aberrant mitochondrial activity, altered calcium homeostasis, and abnormal mitochondrial function in 3xTg-AD mice [52,53,54,55] and APP/PS1 mice [56], two mouse models of AD. These findings support that P-tau is responsible for mitochondrial dysfunction and synaptic damage in AD [35]. Brain tissue collected after death from AD patients at various disease stages, including AD patients who displayed cognitive loss, control subjects without AD, APP, APP xPS1, and 3xTg-AD mice, and control subjects, were used in studies to ascertain the connection between P-tau and Aβ. Also, the study investigated the relationship between monomeric and oligomeric Aβ and P-tau using immunological histology, double-immunofluorescence, and postmortem AD brains. In neurons from AD patients, it was discovered that the monomeric and oligomeric forms of Aβ interact with P-tau. Furthermore, as AD advanced, these meetings happened more frequently. The position of the two proteins was revealed by double-labeling assays of monomeric and oligomeric Aβ and phosphorylated P-tau, revealing that Aβ and P-tau interact more strongly as AD advances. Based on these combined data, the hopeful hypothesis is that A interacts with inappropriately phosphorylated proteins, that this interaction harms synapses and neuronal structure and function, and that this damage results in cognitive impairment in AD patients were formed. Overall, these studies offer solid evidence that in brain tissue from AD patients, hyperphosphorylated tau, is connected to cellular changes primarily linked to mitochondrial dysfunction and synaptic impairment [50].

## 4. Synaptic Loss in AD

Synaptogenic loss is a prevalent and early sign of AD, and the degree of synapse loss is highly linked with dementia. According to some research, AD is an extreme and accelerated form of age-related memory decline [57]; once this accelerated process is initiated, it takes on a pathogenic profile that is not present in healthy aging. Many studies support Aβs’ physiological function in normal synaptic transmission. When synaptic activity increases, secretase activity in organotypic hippocampal slices, the resultant Aβ peptides suppress excitatory transmission through AMPA (α-amino-3-hydroxy-5-methyl-4-isoxazolepropionic acid) and NMDA (N-methyl-D-aspartate) receptors, suggesting a role for Aβ in homeostatic plasticity [58].

Synaptic terminals actively carry impulses between neurons and process information in healthy, undamaged synapses [26,54]. However, in old people and AD patients [59,60], intact synaptic terminals showed alterations that cause cognitive loss. Not all brain areas are damaged equally by AD; the cerebellum, for instance, is unaffected, whereas the hippocampus is affected. A study examining the loss of synaptic connections in the cerebellum and hippocampus found no statistically significant variations in the cerebellum’s synapse-to-neuron ratio between people without AD, nonelderly AD patients, and elderly AD patients. However, in samples from the hippocampus taken from both old AD patients and those without AD, the ratio of synapses to neurons decreased by more than 50% [60,61,62].

These results suggest that the damaged brain area is the only one where synapses are lost in AD [63]. Several morphological and ultrastructural studies discovered a 25–30% reduction in the cortical synapses of AD patients and a reduction of 15% to 35% in synapses per cortical neuron. The quantity of Aβ plaques and NFTs may not be as highly correlated with cognitive decline in AD patients as synaptic loss is [52,53]. Rab3a, synaptotagmin, and other presynaptic vesicle proteins were shown to be lower in non-demented control adults [26,54,60]. These findings imply that the loss of synapses and synaptic proteins may be limited to areas of the brain that have already been impacted by AD, along with the possibility that membrane-bound, presynaptic, and postsynaptic proteins contribute significantly to the emergence of AD. Solvable Aβ, believed to be located at synaptic terminals, has been linked to this loss of synapses and synaptic proteins. Before neuronal death in AD patients, axonal degeneration and decreased mitochondrial axonal transport seem to coexist with the loss of synapses and synaptic proteins [55,62,64]. Many research teams have found that A accumulates at synapses in AD neurons. Our results strongly suggest that synaptic degeneration and synaptic functional failure are primarily caused by mitochondrial dysfunction and abnormalities in AD neurons.

### 4.1. Mitochondrial Dysfunction and Defects in AD

The mitochondrion is a crucial organelle for calcium homeostasis and neuron metabolism. The synthesis of ATP, lipid biogenesis, control of reactive oxygen species (ROS), and calcium clearance are essential biological functions that mitochondria perform [65]. Furthermore, mitochondria are predominately dynamic and can merge, extend, and move down microtubule tracks to ensure they are dispersed to the periphery of neuronal cells. Imped neural development to diverse neurodegenerative disorders, mitochondrial malfunction, and altered potency are seen in an array of circumstances. The role of mitochondria in axon branching, synaptic operations, calcium control with the ER, glial cell functionality, and neurogenetic behavior has been demonstrated to significantly impact AD [66].

#### 4.1.1. Role in the Loss of Neuronal Plasticity and Synaptic Plasticity

The enormous energy needs of neurons, which are highly polarized cells, are met mainly by mitochondria. Mitochondria adjust in correspondence to altered neuronal energy states to support energy balance and nervous system function. Modifications in the form, function, and position can be seen because of this adaptation, also known as mitochondrial plasticity [56]. The synapse, where mitochondria play a crucial role in pre- and postsynaptic processes, is the neurons’ principal site of energy consumption. Mitochondria may play significant roles in managing key neuroplasticity processes, such as brain differentiation, neurite protuberance, neurotransmitter deliverance, and dendritic remodeling, by producing energy (ATP and NAD^+^), controlling subcellular Ca^2+^ and maintaining redox homeostasis [65]. The presynaptic terminals and the axons’ length contain mitochondria, which react to electrical activity and activate growth factors and neurotransmitter receptors [66]. The mitochondria may influence the propensity of neural stem cells to self-proliferate, a characteristic of all stem cells [56].

#### 4.1.2. Mitochondrial Dynamics in Axonal Transport

It is essential to emphasize the immediate relationship between the transport system and the mitochondrial fusion/fission machinery. The RhoT/Trak complex physically interacts with mitofusins (MFN1 and MFN2). The anterograde and retrograde transport significantly decreases when MFNs are inhibited in vivo and in cultured neurons. Transportation within the mitochondria has also been linked to the fission protein named dynamin-related protein 1 (DRP1). Both in vitro and in vivo, DRP1 function inhibition impairs mitochondrial communication associated with dendrites in Purkinje cells [67]. According to extensive scientific expeditions, DRP1 is also crucial for dispersing mitochondrial neuro-termini coordinated with dopaminergic neurons, as it interconnects with the dynein–dynactin complex to modify dynein-based retrograde transport [67].

#### 4.1.3. Mitochondrial Biogenesis

Mitochondrial biogenesis is crucial for maintaining mitochondrial homeostasis as it resembles the assembly of mitochondrial protein and mitochondrial transcription factor A (TFAM), which promotes the transcription and replication of mtDNA; these are some factors that control mitochondrial biogenesis [67]. Peroxisome proliferation activator receptor gamma-coactivator 1 (PGC-1), the principal regulator of mitochondrial biosynthesis, controls the expression of NRF 1, NRF 2, and TFAM [68]. PGC-1, NRF 1, NRF 2, and TFAM expression levels are considerably lower in AD hippocampus tissues, indicating diminished mitochondrial biogenesis [67,68].

#### 4.1.4. Mitochondrial Functions

It is known that mitochondria produce 90% of cellular ROS. Oxidative stress (OS), which results in oxidative damage that affects several cellular components, including lipids, DNA, and proteins, is caused by an abnormality in the balance between the making and breaking of mitochondrial reactive oxygen species (mtROS) [69]. This imbalance is caused by an excess production of ROS and/or a decrease in antioxidant defense activity. Nicotinamide adenine dinucleotide phosphate (NADPH) oxidases (NOX) and other enzymes such cyclooxygenases (COX), lipoxygenases, xanthine oxidases, and cytochrome P450 enzymes are the principal producers of mitochondrial ROS inside the mitochondria [69]. Furthermore, the electron transport chain is inherently dripping; even in the physiological environment, 0.2–2% of the negatively charged particles produced by the pulmonary network are not intertwined to the resemblance of ATP but instead play a major role in the premature interaction of oxygen, which results in the production of superoxide anion (O_2_) or hydrogen peroxide (H_2_O_2_) [70].

The mitochondrial electron transport chain’s complex IV (cytochrome c oxidase, COX) is the final oxidative phosphorylation complex and is particularly susceptible to AD [71]. Although COX deficiencies in AD are well known, the genetic link between COX-related genes and AD has recently been established [71], which extensively reported that AD patients exhibit deficiencies in the complexes of the mitochondrial respiratory chain, particularly those that reduce the effectiveness of complex IV. When focusing on the fusion and fission processes, it is essential to note that these are primarily controlled by proteins from the family of big GTPases called dynamin-related proteins [67]. These proteins drive mechanical motion on biological membranes by hydrolyzing GTP. DRP1 physically interacts with several adaptor proteins, including mitochondrial fission factor (MFF), mitochondrial dynamics proteins 49 and 51 (MID49, MID51), and mitochondrial fission 1 protein (Fis1), to migrate from the cytosol to the outer mitochondrial membrane (OMM) [68]. 

#### 4.1.5. Effects of Aβ and Tau on Mitochondrial Functions

**Figure 2:**
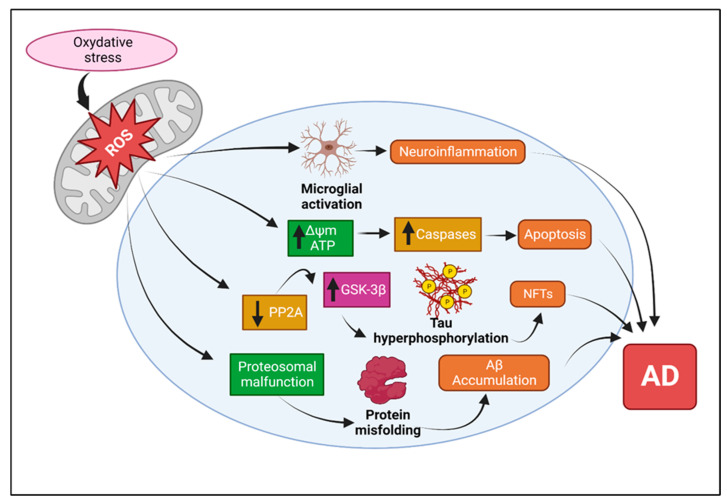
Pathways leading to AD because of oxidative stress and protein misfolding. This diagram illustrates the cascade of events starting with oxidative stress, characterized by the overproduction of ROS. This triggers neuroinflammation and activates microglia, leading to mitochondrial dysfunction (as indicated by decreased ATP levels). The process involves the increased activity of GSK-3β and decreased activity of PP2A, contributing to the hyperphosphorylation of tau proteins. Consequently, there is an accumulation of NFTs and Aβ plaques, which are hallmarks of AD. This sequence of events leads to proteasomal malfunction, further exacerbating protein misfolding and ultimately causing neuronal apoptosis. These interconnected pathways culminate in the development and progression of AD (created with BioRender.com).

In neurons, the secretases cleave the amyloid precursor protein (APP), releasing fragments of Aβ42 [9]. Aβ42 fragments assemble into insoluble extracellular fibrils of neurotic plaques, leading to NFTs. Nevertheless, several molecular, genetic, and contradictory clinical associations have been found. Tau, a key microtubule-associated protein, is vital for the functioning of neurons. The proline-rich region of tau interacts with the microtubule surface, which helps to stabilize the microtubules [72]. Microtubule dysfunction is caused by the non-equilibrium of tau binding to the microtubules, which causes tau to aggregate and fibrillate [9]. The movement of axons depends heavily on the microtubule network. Microtubule disruption from this probably causes aberrant axonal transport and synaptic dysfunction [9]. The ability of signaling molecules, trophic factors, and vital organelles like mitochondria to move through axons is made possible by tau’s substantial impacts on the microtubule network. As a result, tau supports essential cellular regulatory and structural processes [72] (Figure 2).

### 4.2. Endoplasmic Reticulum Stress

Endoplasmic reticulum (ER) stress is believed to play a significant role in AD development. This is primarily due to neuronal malfunction and cell death caused by the piling up of protein misfolding and interference with intracellular calcium homeostasis [73]. Presenilin1 and the amyloid precursor protein (APP) exhibit elevated ER stress responses, according to several investigations (PS1). According to several studies, PS1 controls the homeostasis of ER calcium. Sarco/endoplasmic reticulum Ca2-ATPase, a protein that moves calcium from the cytosol to the ER lumen, and ER-associated calcium channels like the inositol trisphosphate receptor and ryanodine receptor are all affected by PS1. PS1 mutations linked to familial AD (FAD) change the calcium transfer activity of the protein. Endoplasmic reticulum (ER) cytosolic calcium concentration changes are a resilient promoter of ER stress [74].

## 5. Hypometabolism of Glucose in AD

Glucose is the only resource that can pass through the BBB and support typical neural activities. Studies have found that AD patients’ and animal models’ peripheral tissues, including their brains, have impaired glucose uptake and metabolism [75]. Before AD pathogenesis, changes in glucose metabolism happen when oxidative damage builds up. Early-stage AD patients have significantly reduced glucose absorption, which suggests that the condition is preceded by altered glucose metabolism and elevated steady-state glucose concentrations [76]. In addition to facilitating glucose metabolism and influencing tau protein and Aβ processing, insulin in the brain is essential for learning, cognition, neurite growth, and other developmental processes. Impaired insulin signaling causes PI3K activity to decline, which lowers Akt activity, which is necessary for neuronal survival, plasticity, and metabolism. Increased GSK3β activity also encourages tau phosphorylation and Aβ accumulation [75]. When set alongside age-matched controls, AD patients show drastic and insignificant brain insulin levels, IGF-I, and IGF-II receptor [76]. A pre-symptomatic sign of AD called glucose hypometabolism is frequently found alongside early Aβ pathology [77]. Due to its close ties to the majority of the critical AD risk factors, glucose hypometabolism is thought to have contributed to the beginning of sporadic AD. It is also seen in AD patients approximately 20 years before the start of clinical outcomes [77]. It happens in people with amnestic mild cognitive impairment (aMCI), is primarily conceived to actualize a prodromal stage of AD, and is also evident in affected individuals. Additionally, in aMCI patients, glucose hypometabolism may serve as an accurate prediction marker for the emergence of AD [78]. It is not evident that abnormality in glucose metabolism can have a cascade of poisonous effects, since glucose utilization underpins essential brain functions like energy supply and antioxidant defense [77,78]. As such, disturbances in glucose metabolism likely characterize a major underlying cause of ailment onset and maturation [76]. However, the precise origins and effects of AD-associated glucose hypometabolism have been shrouded with unknown facets to this point, impeding the search for a cure.

## 6. Mitophagy and Autophagy Dysregulation in AD

Precise mechanisms regulating organelle and protein quality are needed to maintain neuronal structure and functionality. Autophagy and mitophagy are, therefore, involved in neuronal homeostasis. Functional flaws in removing and recycling intracellular components are critical characteristic features of AD. The pathological phenotypes of AD may be brought on by compromised activity in several cellular pathways. One of the defining characteristics of AD is mitochondrial dysfunction. Mitophagy is a crucial method for regulating the quality of mitochondria, and AD is associated with poor mitophagy [79].

Autophagy, derived from the Greek words “auto” (self) and “phagy” (eating), is a cellular quality control mechanism that selectively or non-selectively clears damaged proteins, nutrients, or cell organelles. While it is active at the basal level under normal conditions, autophagy can also be activated in response to various cellular stresses, such as toxic stimulation, nutrient deprivation, oxidative stress, DNA damage, and protein aggregation [26,50]. This process eliminates cellular products that can cause cytotoxicities, and the degradation products are used for protein synthesis and energy production. Autophagy can be categorized into three classes: micro-autophagy, macro-autophagy, and chaperone-associated autophagy (CMA). Micro-autophagy and CMA involve the direct engulfment of cytoplasmic cargo into the lysosome. In contrast, macro-autophagy involves the formation of an autophagosome that engulfs organelles and cytoplasmic components before fusing with lysosomes to form an autolysosome for digestion [79]. AMP-activated kinase (AMPK) activation and cellular energy deprivation promote transcription factor EB (TFEB), while the repression of mechanistic depletion also contributes to this process. TFEB facilitates the transcription of autophagy-related genes (ATGs) and lysosome-related genes. AMPK phosphorylates and activates Atg/Unc52-like kinase 1 (ULK1), further phosphorylating Beclin1, a class III PI3K complex I (PI3KC3) component. This phosphorylation event triggers the formation of the phagophore membrane. The ULK1 complex also phosphorylates ATG9, promoting the recruitment of PI3P-binding proteins and the ATG12-5-16L complex. These interactions enhance the elongation of the phagophore membrane, the lipidation of LC3, and the recognition of target proteins. After the development and maturation of the autophagosome, it fuses with the lysosome to form an autolysosome. Enzymatic hydrolysis facilitates macromolecule degradation within the acidic lumen of the autolysosome [79]. Defective autophagy has been observed in AD, with abnormalities in mitophagy, excessive oxidative damage, and mitochondrial malfunction. Autophagy dysregulation is considered a characteristic of AD, and impaired fusion of autophagosomes and lysosomes leads to the accumulation of vacuoles in AD brains and transgenic mice models. Further research is necessary to understand AD’s molecular pathways underlying reduced autophagy activity [79].

**Figure 3:**
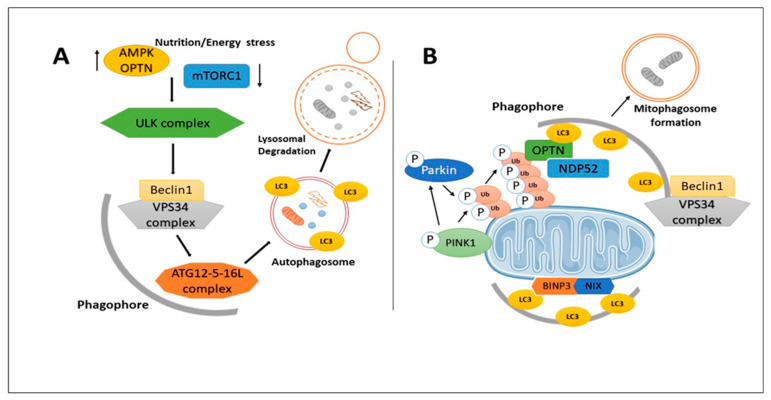
Diagram illustrating the routes for autophagy and mitophagy. (**A**) In response to nutrient or energy stress, AMPK is activated, and mTORC1 is suppressed, which increases ULK1 complex activity and stimulates the creation of the VPS34 and ATG5-12-16L complexes, which, in turn, stimulates the production of phagophores and autophagosomes. (**B**) Depolarization of the mitochondria stabilizes PINK1 and stimulates PINK/Parkin signaling, which increases OMM’s phospho-ubiquitin conjugation. Mitophagy receptors like OPTN and NDP52 identify the polyubiquitin chain, which promotes mitophagosome formation (created with BioRender.com).

Mitophagy is a specific type of autophagy that eliminates damaged mitochondria from the cell, promoting cellular health by reducing the accumulation of defective mitochondria [30]. The proteins PTEN-induced kinase 1 (PINK1) and Parkin play crucial roles in controlling the process of mitophagy. Mutations in PINK1 and Parkin have been associated with neurodegenerative disorders such as AD [80,81,82]. Three main methods can trigger mitophagy in response to mitochondrial damage: ubiquitin-mediated mitophagy, outer mitochondrial membrane (OMM)-receptor-mediated mitophagy, and lipid-mediated mitophagy. The PTEN-induced PINK1-Parkin pathway is the most well-understood mechanism of mitophagy. When mitochondria are damaged, PINK1 stabilizes at the OMM, attracting Parkin. Ubiquitin-binding proteins such as optineurin (OPTN), p62, NDP52, and NBR1 recognize Parkin-ubiquitylated proteins and recruit mitochondria for autophagy [83]. However, recent studies have shown that p62 and NBR1 may not be necessary for Parkin-mediated mitophagy [84]. Several proteins, including Fis1, Drp1, Miro, Opa1, OPTN, Ubiquitin, PINK1, Parkin, and BNIP3, have been identified as essential players in mitophagy. In AD, mitophagy becomes less effective as the disease progresses due to decreased lysosomal system efficacy. Impaired mitophagy in AD is associated with reduced PARK2 levels, inadequate vesicle induction, the accumulation of PINK1, and depolarized mitochondria. Tau and Aβ proteins have also been implicated in the impairment of mitophagy in AD [85,86]; see Figure 3.

## 7. Nano-Based Theranostics in AD

The term theranostic was created by fusing the concepts of treatment and diagnostics. In this new era of medicine, illnesses are diagnosed and treated concurrently or sequentially using a combination of medications and/or procedures. A significant advancement in modern medicine is the capacity to obtain a diagnosis and deliver treatment all in one package. In addition to providing the chance to save time and money, doing so may also allow one to avoid unfavorable biological impacts if these tactics are used separately [87].

Considering the nervous system, specifically the brain, the BBB shields the neuronal “milieu” from external substances, maintaining chemical equilibrium in neuronal circuits and synaptic transmission. Endothelial cells residing inside cerebral capillaries, which make up the barrier, form the primary point of contact associated with the blood and the cerebral system [88]. As a result, pharmaceuticals, including most small molecules, seldom penetrate or leave the BBB [89]. Moreover, current drug delivery technologies cannot offer adequate cytoarchitecture rehabilitation and interrelations, which tend to be essential in AD recovery. As a result, multiple attempts have been implemented over the last 10 years that could address this issue by generating multiple techniques to ease medicine transfer through the BBB [88]. Nanotechnology can improve these drawbacks by offering new carrier-based platforms that target selective domains and release medications on demand, thereby expanding the reach by dodging the BBB. Consequently, NPs may overcome the physiological barriers by themselves. They can be explicitly functionalized to acquire efficient penetration considering their diverse physio-chemical features, such as high stability, increased bioavailability, and the capacity to include hydrophilic and hydrophobic moieties [90,91].

As is well known, drugs can use one of two paradigms to enter the brain quickly: (i) the molecular paradigm, where drugs are injected in an inactive form and become active by specific enzymes at the target site; or (ii) the polymeric paradigm, where drugs are encapsulated and transported to the target site using vehicles such as polymeric vesicles. While polymeric-based carriers can safely carry drugs to the target site without their loss and achieve maximal bioavailability, the main limiting factor in the molecular method is the exposure of drugs to enzymatic degradation, which minimizes their bioavailability. Thus, creating polymeric nanostructures has been suggested as one of the most secure means of treating the illness [91].

According to recent studies, a contemporary theranostic platform for AD has been made possible by bioengineering nanostructures with the desired functional groups to cover or encapsulate drugs or track agents in a single particle and deliver them simultaneously. Therefore, some potential treatments for this condition include Cerium(IV) oxide (CeO_2_) NPs, which support neuroprotection via the redox pathway, AuNPs, which solubilize Aβ clusters, and DNA nanoparticle conjugates. Nanogels with Aβ anti-assembly alter protein folding patterns, fullerenes, and their derivatives limit oxidative stress and neurotoxicity. All of these substances are employed in pharmacological therapy to lower amyloid levels, including memantine, NPs produced primarily of zinc, vitamin D-binding protein, SNPs, Aβ42 peptides, coumarin, liposomes, and dendrimers, as well as nanospheres formed of selenium and curcumin [92].

In addition, several cells, including those in the CNS, release exosomes, which are tiny vesicles ranging from 30 to 100 nm. They contribute to accumulating proteins like Aβ, tau, and prions in the brain and spreading pathogenic proteins [93,94]. Also, since body fluids may cross the BBB while carrying many genetic components (such as DNA, miRNA, and protein) necessary for neuron function, it is easy to learn about brain health through them [95,96]. Exosomal miRNAs (exo-miRs) can provide accurate information on the many elements of AD according to the disease progression. Exo-miRs are engaged in various crucial CNS processes, including neuronal differentiation, maturation, and functioning of adult neurons [97]. The potential of specific nanosystems, their type, strategy, size, conjugated drugs, target, and main functions are listed in Table 1.

## 8. Nanotechnological Diagnostic Tools

### 8.1. In Vivo Diagnosis

#### 8.1.1. Nanoparticles in AD Diagnosis Using MRI

Using mono-crystalline iron oxide NPs (MIONPs) and ultra-super-magnetic iron oxide NPs (USIONPs) as MRI contrast agents can considerably impact the in vivo detection of AD in transgenic mice models. The simultaneous targeting and imaging of senile plagues are improved by using MIONP and USIONP to target amyloid protein plagues. MRI is the answer to this problem. Amyloid plaques are bonded by a substance called Congo red. Due to their excellent contrast-to-noise ratio, Congo-red-loaded magnetic NPs (CR-MNPs) allow for the precise detection of amyloid protein on MRI [110].

#### 8.1.2. Optical Imaging

Optical imaging techniques are known to have precision and affordability and offer a variety of contrast agents. Fluorescent probes and agents like Alexa-fluor-750-conjugated BAM-10 and fluorescein-labeled 10d5 tag antibodies specific for Aβ. However, most of these tags are usually administered locally since they cannot penetrate the BBB. We explicitly target the AD-related biomarker in the optical imaging detection process. A well-known fluorescent probe and amyloid fibril detection agent is thioflavin Ts. Pleated sheets of Aβ aggregates may be recognized by thioflavin T both in vitro and in vivo, and Congo fluorescence imaging may be used to detect red derivative methoxy-X04, Thioflavin S, fluorescent probes like AOI987, and curcumin-derived CRANAD-2 that binds to Aβ [92].

### 8.2. In Vitro Diagnosis

#### 8.2.1. Biosensor or ELISA-Based Detections

Conventional technologies, such as PET, MRI, and others, are often used to detect AD precisely but are expensive, time-consuming, and unaffordable to the maximum number of individuals. Considering the situation, biosensors are becoming major alternative tools for rapid, economical, and precise AD diagnosis [111]. Therefore, biosensors like immune biosensors, DNA biosensors, and optical and electrochemical biosensors can accurately identify an oligomer of Aβ. These are crucial for the diagnosis of AD. Over time, significant advancements have been made in creating biosensors for identifying the primary biomarkers of AD [112,113].

The exceptional and distinctive characteristics of NPs enhance the electrochemical and optical behaviors of sensors. The fabrication of biosensors for identifying the primary biomarkers of AD depends critically on the coupling of nanomaterials and various sensors. Numerous analytical procedures with diverse features employ NPs extensively. About 1–100 nm in size, the nanoscale scale is distinguished by its distinctive structure, electron, magnetism, optics, catalysis, and biocompatibility [114].

Moreover, point-of-care (POC) biosensors and recently identified biomarkers have increased prominence in enhancing AD detection. Firstly, the preliminary examinations are convenient and hassle-free, often requiring only a few microliters (5–50 μL) of samples (plasma, urine, saliva, or CSF) for assessment without time-consuming preparation. Results are usually provided within 2 to 5 h, and the research is incredibly straightforward, requiring essentially no hands-on effort and no specialized personnel. Due to the high specificity of the potential bioreceptor choices, biosensors may also be constructed to demonstrate excellent performance on complicated fluid analyses. As a result, these can, for instance, show excellent findings employing blood. The gadgets are, therefore, appropriate for detecting and measuring the recently identified AD biomarkers [111].

#### 8.2.2. Electrochemical Biosensors

In this instance, the analyte is detected and quantified by detecting the changes in conductivity, impedance, and redox reactions [111]. It has been extensively reported that voltammetric measurements, which assess the system currently as a function of an applied potential, can find AD biomarkers. An enzymatic biosensor for acetylcholine (ACh) ACh detection in plasma was created by Moreira et al., employing a very porous gold electrode functionalized with acetylcholinesterase (AChE) [115]. The interaction was assessed using square wave and cyclic voltammetry, and the instrument showed a detection limit of about 10 µmol L^−1^. Voltametric methods can be used with signal amplification tactics to reduce the limit of detection (LOD). Using silver NPs functionalized with alkaline phosphatase-labeled antibodies as a signal enhancer in differential pulse voltammetry (DPV) measurements, Zhu et al. described a sandwich-type biosensor for α-1 antitrypsin. The biosensor demonstrated high repeatability and LODs as low as 0.01 pmol L^−1^. Using the sandwich approach, Yu et al. created a sensitive biosensor for measuring soluble Aβ levels [111]. In this instance, gold NPs (AuNPs) functionalized with horseradish peroxidase (HRP) and gelsolin increased the signal. Owing to its high attractive affinity towards Aβ, gelsolin has been exploited as a primary protein immobilized over a nano platform. The biosensor displayed a detection limit of about 28 pmol L^−1^ using the DPV approach.

Another electroanalytical technique extensively researched in creating biosensors for AD is electrochemical impedance spectroscopy (EIS). EIS employs impedance changes to identify the biomarker molecule. The method is well-recognized in the literature because it enables the quick examination of several species with low LOD, high sensitivity, and little expense. An EIS-based detection method for A oligomers was created by Rushworth et al., employing a biorecognition element derived from a piece of the cellular prion protein [111]. Their biosensor demonstrated a linear response and attained a 0.5 pmol L^−1^ detection limit. Esteves-Villanueva et al. created a protein-based EIS biosensor employing tau protein as a biorecognition element to assess tau oligomerization [116]. The proposed biosensor may be utilized to test possible aggregation inhibitors by detecting tau–tau binding.

#### 8.2.3. Optical Biosensors

Several optical biosensors have recently been created to help diagnose and treat AD [111]. For instance, Palladino et al. immobilized specific antibodies onto biosensor chips and utilized SPR to examine the development of Aβ plaques in real-time. The ability to analyze without employing fluorophores, which might affect the aggregation process, is a benefit of this technique. This approach provides a fresh tool for studying aggregation inhibitors and can help create disease-treating medications. Yi et al. created a biosensor employing SPR in 2018 for the genotyping and measuring of apolipoprotein E (ApoE), the main genetic risk factor for AD [111]. Scientists used biotinylated DNA probes corresponding to the ApoE 4 allele sequence to modify Au films. After hybridization, streptavidin was introduced, and an enzymatic cleavage reaction was carried out. The streptavidin could bind to the biotinylated DNA probes for single-base mismatched sequences. However, the complementary sequence hampered the biotin–streptavidin binding, which led to various SPR signals. At femtomolar concentrations, the biosensor was capable of selectively detecting DNA sequences. Doong et al. have created an enzymatic optical biosensor for concurrently evaluating many AD biomarkers [111]. In this instance, fluorescent dyes were immobilized on sol-gel substrates, and the pH shift brought on by the interaction between the enzyme and the analyte stimulated the dyes and produced a signal. The biosensor performed well and had low LODs for glutamate (Glu), Aβ, and ACh. Finally, Zhang et al. created a high photocurrent intensity photoelectrical immunosensor for A detection based on SnO_2_/CdCO_3_/CdS nanocomposites [111]. They noted that the linear range for Aβ was broad (0.1 pg/mL to 100 ng/mL), while the detection limit was low (50 fg/mL).

**Figure 4:**
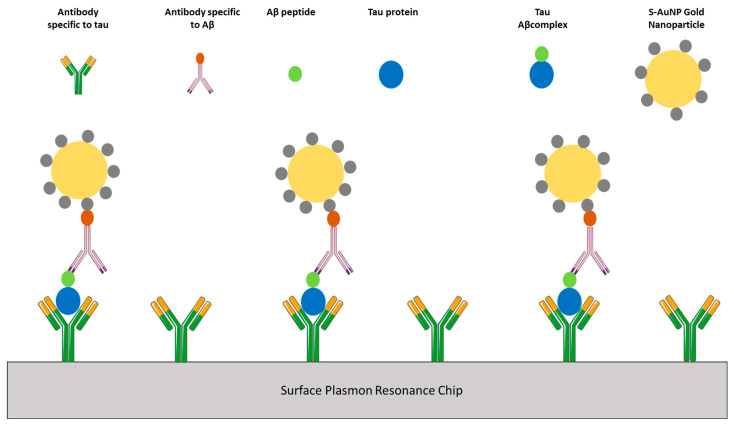
Diagram illustrating how antibodies specific to tau and Aβ work near each other; along with streptavidin-coated gold nanoparticles (S-AuNP) and biotin-coated Aβ-antibody interaction, we can diagnose AD (created with BioRender.com).

Researchers employed Aβ-specific monoclonal antibodies and tau protein-specific monoclonal antibodies. AuNPs were coated with streptavidin, whereas Aβ-primary antibodies were biotin-coated. A self-assembled monolayer of COOH operated the SPR surface- and OH-thiols [114]. The primary antibodies directed against the tau proteins were initially immobilized to enable them to connect covalently with carboxylic groups. A patient’s CSF sample was utilized on the sensor surface to allow the tau-Aβ complex to bond with any available tau-antibody. The SPR surface was made functional. The biotin-coated Aβ-primary antibody was incorporated, and S-AuNP was put into the chip. With the biotin-coated Aβ-antibody, the streptavidin-coated AuNPs will bind precisely since biotin is known to interact with streptavidin. The non-covalently-attached particles were ultimately removed, the remaining carboxyl groups were deactivated, and the SPR peak was measured when PBS and aqueous ethanolamine were employed to do so. SPR sensors may be used based on surface plasmon wavelength spectroscopy. The sensor response was represented as a change in the wavelength at which the SPR fall takes place, commensurate with an alteration in the sensor’s refractive index brought on by the molecules adhering to its surface [117,118] (Figure 4).

Moreover, AuNPs have been used widely in AD biological analysis as they are non-toxic and simple to operate. They also feature a high surface volume ratio, strong electrical conductivity, stability, and biocompatibility [114]. The significant characteristics of AuNPs are their stability and capacity to attach to biological elements [114]. For the created sensors for the detection of Aβ, AuNPs can bond with Aβ [119]. AuNPs have also been shown to be desirable materials for detecting various analytes and studying the interactions of metal ions and amyloid peptides due to their optical and electrochemical selectivity and biocompatibility. AuNPs can also boost the signal and increase the effectiveness of electron transfer [114]. The development of electrochemical biosensors for detecting biomarkers will benefit from the ability of AuNPs to amplify signals [117]. Different AuNPs have functions in the sensors, and the sensors are crucial for the diagnosis of AD. Magnetic NPs are commonly used in bioanalysis because they may function as both a stable support and a method of separation in the system. Moreover, they collect samples using a magnetic field, making them one of the most promising and sensitive sensors [120]. Since CSF reflects metabolic changes in the brain, it is a viable alternative for discovering potential AD biomarkers [121]. As a result, CSF-biomarker-based sensors are the most popular AD diagnostic instrument, offering solid justification for the diagnosis [120]. A bio-barcode amplification test based on ultrasensitive NPs was created by Klein et al. to assess AD soluble biomarkers in CSF at clinically significant quantities. Specifically, the Aβ-derived diffusible ligands (ADDLs) were captured and pre-concentrated utilizing magnetic microparticles to construct the nanostructured platform. The additional detection of the “sandwich” immunoassay different detection was based on the oligonucleotide-modified AuNPs; the system was able to identify ADDLs at extremely low concentrations (even at the level of 10^−18^ moles per liter), significantly increasing the sensitivity of the ELISA test by 6 orders of magnitude. This was achieved by implementing antigens sequestration in solution and the amplification process resulting from massive DNA strands released for each antigen discrimination [120].

Zinc oxide (ZnO) is well known as a semiconductor metal oxide nanoparticle that possesses optical attributes. Nanoflowers provide a larger surface area among all ZnO nanostructures. By leveraging this characteristic, a reagentless biosensor has been designed and created to detect beta amyloids, a defining feature of AD. Glass slide preparation in this technique includes the activation, surface treatment, and electroless deposition of nano-silver (nano-Ag) thin film [122]. After that, ZnO NPs were synthesized and spin-coated on the glass slides [123]. Subsequently, zinc nanoflowers were grown on the seeded substrate (ZnO NPs in 1% acetic acid solution). Then, those treated glass slides were dipped in a growth solution consisting of zinc nitrate hexahydrate and hexamethylene tetramine (HMT) [110]. Only glass (activated glass slide), Substrate-A (ZnO NPs produced on activated glass slide), Substrate-B (nano-Ag coated glass slide), and Substrate-C (ZnO NPs grown on nano-Ag coated glass slide) were then submerged in thioflavin T (ThT) solution and stored at 4 °C for 12 h. Substrates coated with ThT were then dried by air in a dust-free environment [124]. Amyloid is very sensitive to conventional ThT assay. ThT increases the intensity of its emission spectrum when it binds to amyloid fibrils, while non-amyloid proteins do not increase their fluorescence intensity when they attach to ThT. Thus, a proper result can be obtained from the fluorescence absorbance outcome and further processed to decide and start with detailed therapeutic procedures [125].

## 9. Therapeutic Interventions for AD

Therapeutic interventions for AD aim to alleviate symptoms, slow disease progression, and improve the quality of life for individuals affected by this neurodegenerative disorder. With the increasing prevalence of AD and its significant impact on individuals and their families, researchers and healthcare professionals have been actively exploring various treatment approaches and interventions. These therapeutic interventions encompass a range of strategies, including pharmacological treatments, lifestyle modifications, cognitive and behavioral interventions, and ongoing research into emerging therapies, with the ultimate goal of advancing our understanding and management of AD [126].

We will further discuss different nanocarriers that possess a greater role in therapeutics, but first we will look at some of the significant challenges we face in using nanocarriers for brain delivery. Their accumulation in the reticuloendothelial system (RES), particularly in the liver, carries a significant risk. The liver’s RES, which includes Kupffer cells and liver sinusoidal endothelial cells, is highly efficient at capturing and clearing nanoparticles from the bloodstream. This accumulation in the liver can limit the amount of nanocarriers that reach the brain and raises concerns about potential toxicity. To address this issue and enhance brain delivery, researchers have developed several innovative strategies.

***In Situ PEG Coating***

One approach involves applying a transient PEG (polyethylene glycol) coating to liver scavenger cells using a two-armed PEG-oligopeptide. This PEG coating creates a temporary barrier on the surfaces of Kupffer cells and liver sinusoidal endothelial cells, which are responsible for capturing and clearing nanoparticles. When the liver scavenger cells are coated with PEG, they become less effective at capturing subsequent injections of nanoparticles. As a result, these nanoparticles are less likely to be taken up by the liver and can accumulate in their intended target organs, such as the brain [127].

***“Don’t-Eat-Me” Signal Peptides***

Another strategy involves decorating nanocarriers with “don’t-eat-me” signal peptides derived from the CD47 protein. CD47 is a protein that inhibits phagocytosis by signaling to macrophages and other scavenger cells not to engulf the particles. By incorporating CD47-derived peptides on the nanocarriers, these particles can avoid recognition and uptake by liver scavenger cells. This coating forms a protective “mask” around the nanocarriers, reducing their interaction with liver RES and allowing more nanocarriers to remain in the bloodstream [128].

***Enhanced Brain Delivery***

**Figure 5:**
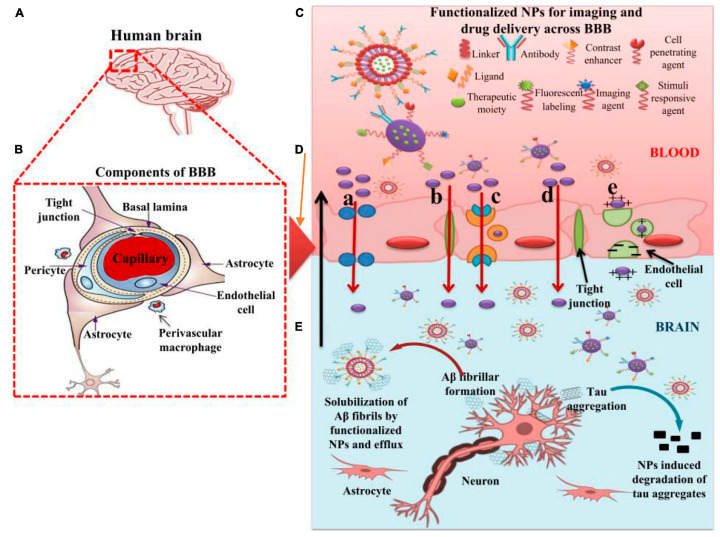
The role of nanoparticles in overcoming the BBB for efficient delivery of therapeutic moieties to treat AD. (**A**) Image of human brain. (**B**) Components of the BBB. (**C**) Functionalized nanoparticles (NPs) for imaging and targeted drug delivery to the AD brain. (**D**) Different pathways of transport (a–e) across the BBB utilized by functionalized NPs. (a) Transport of NPs through cellular transport proteins; (b) transport of NPs through tight junctions; (c) transport of NPs via receptor-mediated transcytosis; (d) transport of NPs via transcellular pathway following diffusion, specifically adopted by gold NPs; (e) transport of cationic NPs and liposomes via adsorption-mediated transcytosis. (**E**) Effect of functionalized NPs in treating AD via the degradation of tau aggregates and efflux of Aβ fibrils after becoming solubilized by the NPs (reprinted with permission from ref [129] with CC BY 4.0 license Copyright© 2021 Khan, Mir, Ngowi, Zafar, Khakwani, Khattak, Zhai, Jiang, Zheng, Duan, Wei, Wu, and Ji).

These strategies combined significantly enhance the accumulation of brain-targeted nanocarriers. In a model of cryptococcal meningitis, this approach led to a dramatic improvement in the delivery and effectiveness of the therapeutic nanocarriers compared to conventional therapies. By reducing liver uptake and extending the circulation time of the nanocarriers, these strategies enable higher doses to reach the brain, ultimately resulting in better therapeutic outcomes [127,128]. The role of nanoparticles in overcoming the BBB for the efficient delivery of therapeutic moieties to treat AD is described in Figure 5.

### 9.1. Biogenic Nanotherapeutics

These NPs are frequently derived from various biological models or produced similarly utilizing natural techniques. Because they are biocompatible and have a lower probability of rejection, they offer promising avenues for treating AD. By harnessing the power of nanotechnology, these innovative therapies utilize biologically inspired NPs to target and deliver therapeutic agents specifically to the affected brain regions, offering potential precision and efficacy in AD treatment.

Biogenic nanotherapeutics can enhance drug delivery across the BBB, allowing for improved access to the brain and optimized therapeutic outcomes. Moreover, these nanotherapeutic approaches can target key pathological hallmarks of AD, such as beta-amyloid plaques and NFTss, aiming to slow disease progression and mitigate cognitive decline. Ongoing research and development in this field hold promise for novel treatment modalities and the potential to revolutionize AD management by offering targeted and tailored therapeutic interventions [130].

#### 9.1.1. Exosomes

In recent years, exosomes have become the most promising biomarkers for illness detection and a focused drug delivery system for disease therapy [131]. Exosomes offer several benefits over synthetic drug delivery systems like liposomes and NPs due to their endogeneity, favorable pharmacokinetics, unique immunological features, and capacity to cross physiological barriers [132]. Exosomes operate as active messengers in the neurological system and defend neurons against oxidative stress. Exosomes are said to assist in the degradation of Aβ 1–42 [133]. Recent research has demonstrated that adipose-derived mesenchymal stem cells conditioned media enriched with exosomes mediated direct neuroprotection by inhibiting cell apoptosis by targeting PTEN-PI3K/Akt pathway and promoting nerve regeneration and repair [133]. To provide a solution against AD, exosomes were employed by Lvarez-Erviti et al. to carry siRNA to the brain and inhibit the production of BACE1, a key secretase responsible for the production of amyloid peptides. Exosomes synthesized in neurons were also shown to break this peptide [134]. Another study team looked into how exosomes may help curcumin cross the BBB more easily through receptor-mediated transcytosis. Additionally, curcumin-loaded exosomes increased the activity of the AKT/GSK-3 pathway, which prevented tau phosphorylation and improved both in vitro and in vivo neuronal death prevention [135].

Along with these herbal ingredients, other substances transported into plasma exosomes were examined. Exosomal formulation enhanced therapy for the symptoms of AD by avoiding tau pathology more effectively than the substances in free form. Quercetin is one such example of an herbal chemical. It is a flavonoid with anti-inflammatory, antioxidant, and neuroprotective properties [135]. All these need more detailed work and progress shortly to come to a proper solution that will benefit individuals.

#### 9.1.2. Liposomes and Lipid Nanoparticles

Liposomes are a kind of therapeutic carrier NP that are highly flexible and biocompatible [136]. They may readily be functionalized to engage with specific molecular targets and can integrate hydrophilic pharmaceuticals in the aqueous pore or hydrophobic chemicals in a lipid layer [137]. With advancements in nanotechnology, we can now generate the BBB by targeting surface-modified liposome ligands to cross the BBB via transcytosis. Recently, other than conventional liposomal delivery systems such as liposomes conjugation with ligands like Tf and Lf to cross over the BBB via receptor-mediated endocytosis [138], cell penetration peptide (CPP)-modified liposomes that target different amyloid markers and help in inhibition of their expression and magnetic liposomal delivery system have been explored to improve brain drug delivery efficiency [139].

Scientists have identified multifunctional liposomes. They have associated a curcumin derivative and a BBB transport mediator (anti-transferrin antibody (TrF)). These conjunctions have a higher affinity for amyloid deposits and can be a potential therapeutic option [140]. Another research group has bi-functionalized those previously derived liposomes with mApoE and phosphatidic acid (PA). mApoE enhances the crossing ability, and PA is a high-affinity ligand. In vivo, studies regarding this have shown a reduction in amyloid plaque load. This mApoE-PA-liposome complex will be an attractive avenue in AD treatment [103].

Other than this, recent studies have shown that lactoferrin-mediated berberine nano-liposomes have been tested on mouse models. To establish AD, the mouse was injected with amyloid-beta 1–42 into the lateral ventricle of the mouse. The aforementioned liposomes have been shown to inhibit AChE activity and apoptosis in the hippocampus cells. They even reduced tau over-phosphorylation. This has enabled improved mouse behavior. These findings impact the therapeutics of AD and prove to be a path maker in future effective treatment [141].

#### 9.1.3. Biopolymers and Nanoformulations

Polymeric NPs are considered the most desirable because of their biodegradability, biocompatibility, long shelf life, and sturdiness during storage, which could offer a regulated and continuous load release. The natural polymers chitosan, sodium alginate, and gelatin, as well as synthetic polymers like polylactic acid (PLA), polyglycolic acid (PGA), poly butyl cyanoacrylates (PBCA), acid (PLGA), polycaprolactone (PCL), and poly lactic-co-glycolic acid, can be utilized to employ the biopolymer NPs for drug administration in the CNS [142].

Biopolymer NPs stand out among drug delivery methods due to their nanometric size and potential for being specially designed for targeted distribution and controlled release. Passive transport and active transport are the two transport methods that these biopolymer NPs use. They frequently have PEG coatings. The most popular ligand up to this point is PEG. It offers stability by causing NPs to experience a sheath effect, which enables them to avoid the immune system’s onslaught [143].

Scientists have experimented with and tested various models to comprehend their workings and minute impacts. Mittal et al. gave estradiol-loaded PLGA NPs to AD-modeling rats to simulate a post-menopausal situation. Post-menopausal AD is thought to be a risk due to low estradiol levels. In the rat illness model, they discovered that PGLA NPs successfully suppressed the expression of Aβ42 [144].

In a study, a novel therapeutic approach was tested for AD using a specialized delivery system called a polyplex micelle. This micelle was designed to carry mRNA encoding a single-chain variable fragment (scFv) antibody specifically targeting Aβ proteins, which are known to accumulate and form plaques in the brains of AD patients [145].

The polyplex micelle efficiently delivers the mRNA into cells, where it instructs them to produce the anti-Aβ scFv antibody. This antibody is engineered to bind to and neutralize Aβ proteins, thus reducing their harmful accumulation [145].

The therapy was administered to mice with acute amyloidosis, a model that mimics the severe amyloid plaque deposition seen in AD. Following the intracerebroventricular injection of the polyplex micelle containing the mRNA, there was a significant reduction in fibrillary Aβ levels, indicating a decrease in amyloid burden in the mice’s brains. This result demonstrates the potential of this mRNA-based approach to reduce amyloid plaques and potentially offer a new strategy for treating AD [145].

Researchers also experimented using a polymeric micelle—tiny, spherical nanoparticles about 45 nm in diameter—that was modified with glucose ligands. These glucose modifications help the micelle cross the BBB more effectively.

The micelle was loaded with antibody fragments known as 3D6-Fab. These fragments are designed to target and bind to soluble amyloid beta (Aβ_1–42_) proteins, which are implicated in AD. When injected intravenously into AD model mice, the glucose-modified micelles successfully navigated through the BBB and entered the brain.

Inside the brain, the micelles released the 3D6-Fab antibody fragments. These fragments are then bound to the soluble Aβ_1–42_ proteins, preventing them from aggregating into harmful plaques. Additionally, the antibody-Aβ complex was taken up and cleared by astrocytes, a type of brain cell that helps maintain brain health. This approach not only inhibits the formation of toxic Aβ aggregates, but also facilitates their removal, offering a promising strategy for treating Alzheimer’s disease by targeting the Aβ pathology [146].

In a recent study, researchers developed a polyplex micelle that co-loads both Cas9 mRNA and single-guide RNA (sgRNA) to achieve efficient genome editing in the brain. This micelle was used to deliver the components necessary for CRISPR/Cas9-based genome editing directly into the brain tissue of mice through intraparenchymal injection.

Cas9 mRNA encodes the Cas9 protein, which acts as molecular scissors to cut DNA at specific sites, while sgRNA guides the Cas9 protein to its target sequence in the genome. By co-loading both Cas9 mRNA and sgRNA into the same micelle, the researchers ensured that both components were delivered simultaneously to the brain cells. This approach led to efficient genome editing across various types of brain cells, including neurons, astrocytes, and microglia.

The study found that this co-delivery method resulted in more effective genome editing compared to delivering Cas9 mRNA and sgRNA separately. When Cas9 mRNA or sgRNA was delivered individually, the editing efficiency was lower, likely due to suboptimal timing or insufficient amounts of one component relative to the other [147].

#### 9.1.4. Phytocompound-Conjugated Systems

Emerging nanotechnological interventions have enabled specific and sustained drug release. Phytocompounds encapsulated in polymeric substances can treat AD more safely. Their ability to overcome the BBB is a significant advantage. Several studies have been conducted in this regard. The encapsulated formulation of curcumin in poly n-butyl cyanoacrylate significantly impacts neuroblastoma cells. These compounds inhibit Amyloid-beta 1–42-induced toxicity. Therefore, their role in AD therapy is crucial [148]. Scientists have also tried curcumin-loaded PLGA conjugated to Tet-1 protein and found that it influences curcumin uptake by neuronal cells compared to plain curcumin [149]. Curcumin is essential for the suppression of AD. Thus, this assembly can function as a possible anti-Alzheimer drug. Ligustrazine phosphate is a phytochemical isolated from the Chinese herbal medicine Haoben Chuanxiong when loaded onto liposome and administered transdermally, resulting in behavioral changes in the disease profile of the affected personnel. This suggests that the formulation functions and impacts the treatment [150]. In addition, curcumin-coated nanoliposomes have been shown to affect anti-fibrillogenic activity, which helps recover from the disease [151]. Another research group has attempted to encapsulate Vitamin E (tocopherol) in polyethylene glycol (PEG)-based nanospheres. This assembly could prevent A-beta-induced ROS formation in human neuroblastoma [105].

Along with curcumin, other plant-derived products such as resveratrol, piperine, epigallocatechin-3-gallate (EGCG), gallic acid, ferulic acid, thymoquinone, ginkgolides, punicic acid, ginsenoside, coumarin, rosmarinic acid, berberine, hesperidin, and retinoic acid are used as AD therapeutics. These phytochemicals have better functionality and act more intensely when loaded onto different nanocarriers. The cost of phytochemicals is comparatively low, and patients become less vulnerable to side effects. With the advent of technology, therapeutics have become easy to access for all types of people, and future research will continue to improve human health [152].

### 9.2. Metallic and Inorganic Nanosystems

Despite their exceptional thermal, mechanical, electrical, and optical capabilities and distinctive structure, they can be used for quick application. Still, for a long-term solution to the drug delivery domain, they are not considered extraordinary. The common thread they possess is their neurotoxic consequences like cell growth inhibition, the promotion of reactive oxygen species generation, the promotion of lipid peroxidation, and the loss of astrocyte function. Here, we will discuss some of the well-known systems used in the therapeutics of AD [153].

#### 9.2.1. Carbon Nanotubes (CNTs)

In the field of nanomedicine, carbon nanotubes are the most widely used carbon-based nanomaterial for the delivery of medications, hormones, and enzymes, gene therapy, and tissue engineering, as well as for biosensors, nanoprobes, and nanorobots [152]. CNTs are elongated hollow cylindrical nanostructures made of sheets with diameters ranging from 1 nm to 10 nm. In the case of CNTs, the sheets contain carbon as the atom and, when rolled, are transformed into nanotubes. There are two types of CNTs: (a) single-walled nanotubes (SWNTs), which consist of a single carbon sheet wrapped into a central tubule, and (b) multi-walled nanotubes (MWNTs), which have many graphite layers around a core tubule [115].

These nanotubes possess exceptional qualities that make them promising candidates for efficient drug delivery systems. They exhibit a high drug-loading capacity and the ability to traverse challenging biological barriers, such as the BBB. Furthermore, CNTs can facilitate drug transport to the brain via the olfactory route, thereby aiding in restoring normal autophagy and preventing the elimination of autophagic substances. They can transport a wide range of cargo, including drugs, antigens, genetic materials, and biological macromolecules. As a drug delivery system for anti-Alzheimer’s drugs, CNTs hold great potential in overcoming physical barriers like the BBB. However, further extensive research is necessary to establish a solid foundation for developing advanced commercial products based on carbon nanotubes for treating AD [154]. For example, by precisely controlling the dosage, SWCNTs successfully delivered ACh into the brain to treat experimentally induced AD. This approach exhibited a moderate safety range and involved directing the SWCNTs to enter lysosomes, specifically, the targeted organelles involved in the treatment [155].

#### 9.2.2. Dendrimers

These synthetic polymers contain repeating units that are highly branched and arise from a single focal point. They have many exposed anionic, cationic, or neutral groups, which gives them hydrophilic or hydrophobic properties [156]. Their size ranges from 1 nm to 10 nm, and they are radially symmetrical, globular, monodispersed, and homogenous [106]. As they are extremely useful nanocarriers in drug delivery, they can open potential avenues for treating AD [157]. Two main types of dendrimers are found to be used in treating AD. Poly(amidoamine) dendrimers (PAMAM) have higher degrees of drug loading [158], increased physical or chemical interactions between drug molecules and tertiary amine groups, and increased conjugation degree due to many terminal groups. Phosphorous dendrimers contain phosphorous as the leading inorganic group in their structures and are extensively used in drug delivery alone or complexed with other dendrimers [157]. The significant benefits of dendrimers are their adaptability, biocompatibility, and ability to load pharmaceuticals into the core, surface, and nano-size. Dendrimers conjugated with ligands can pass through the BBB and improve the uptake of medicines in the brain’s target regions [159].

Recently, it has been seen that oxidative stress and the activation of glial cells can induce inflammatory responses in AD. Therefore, a ROS-responsive dendrimer conjugated with peptide had been designed by Liu et al., which reduced ROS levels, promoted Aβ phagocytosis, and reduced inflammation in the AD microenvironment by targeting and delivering peptides to the nuclear factor (erythroid-derived 2)-like signaling pathways [158]. G4 poly (propylene imine) (PPI) dendrimers modified with histidine maltose shell NPs were delivered to AD mouse models, resulting in memory protection [160]. Aβ peptide was also said to attach to sialic acid residues on the cell surface, leading to neurotoxicity [161]. Thus, removing sialic acid residues or mimicking the cell surface with sialic acid-conjugated dendrimers can be a potential therapeutic strategy. PAMAM containing 32 (G3) and 64 (G4) terminal groups and conjugated with sialic acid residues had been shown to reduce neurotoxicity in animal models. Similar results have been found by loading PAMAM with an anti-AD drug like memantine hydrochloride [162].

#### 9.2.3. Quantum Dots (QDs)

QDs are a highly stable class of nanoscale semiconductors with high quantum yield, absorbency, and photobleaching resistance [163]. These can diagnose and treat mitochondrial dysfunction in ADs [164,165,166]. This was verified by Hoshino et al.’s experiment, which prepared mitochondrion-targeted QDs called Mit-8-QD that emitted red fluorescence compared to non-mitochondrion targeting controls. This red fluorescence was emitted on co-localization with mitochondrion. Also, a TPP (triphenyl-phosphonium bromide)-modified molybdenum QD (TPP-MoS_2_-QD) was designed to penetrate the BBB and target the mitochondrion. The co-localization levels of TPP-MoS_2_-QDs were significantly higher than those of control MoS_2_-QDs without TPP [167]. It was also found that this type of QDs reduced Aβ mediated ROS and prevented the disappearance of OMM and mitochondrial cristae caused by Aβ [168]. Moreover, TPP-MoS_2_-QD reduced neuronal death in AD-infected mice cells compared to control cells (given no TPP-MoS_2_-QDs), thus proving to be a positive theranostic approach in AD [164].

Other strategies to treat AD, besides targeting mitochondria, have also been explored. In a recent experiment, selenium quantum dots (SeQDs) were prepared with high BBB activity and a high cellular uptake rate. In vitro experiments revealed that these QDs interfered with transforming Aβ monomers into aggregates, thus preventing their accumulation. In vivo experiments showed that SeQDs possessed antioxidant activity, improved mitochondrial dysfunction, and inhibited abnormally phosphorylated tau protein accumulation [169].

Recently, another group of scientists studied a new class of NPs called graphene quantum dots (GQD) derived from the flowers of *Clitoria ternatea*. These ctGQDs are special. They have a shallow cytotoxic profile. They are biocompatible and can show improved AChE inhibition ability. Due to their small size, they can cross the BBB and transport drug molecules into the brain without impacting typical systemic regulations [103].

#### 9.2.4. Metallic Nanoparticles and LSPR-Based NPs

Oxidative stress is a significant factor in the progression of AD, while certain metals can lead to neurotoxicity. Metal NPs (MNPs) like Ag, TiO_2_, and ZnO can migrate and accumulate in the brain, causing permanent damage [170]. Selenium NPs (SeNPs) have been suggested to possess neuroprotective effects against AD [171]. In a recent study, B6 peptide-coated SeNPs functionalized with sialic acid demonstrated successful crossing of the BBB. EGCG-stabilized SeNPs coated with Tet-1 also showed promise in hindering Aβ aggregation [172]. Another study explored the use of biogenic AuNPs synthesized from *Terminalia arjuna*, a medicinal plant used in traditional medicine. These gold NPs not only inhibited Aβ fibrillation, but also disrupted mature fibrils. Additionally, they significantly inhibited cholinesterases (ChE), a popular strategy for improving AD conditions by increasing ACh levels [173]. Given the positive results of biogenic gold NPs, further research should be conducted on biogenic Se, Ru, ZnO, and other NPs using therapeutic plants. These green chemistry methods are cost-effective and environmentally friendly, providing a platform for synthesizing NPs with diverse properties [174]. The evidence regarding MNPs necessitates more research on their potential applications in AD, including a detailed investigation of their safety and toxicity in the brain [175]. One such method is a metal chelator such as N, N′-1,10-Bis(naringin) Tri-ethylene-tetraamine. It prevents Cu^2+-^-induced Aβ aggregation, making it a potentially effective therapeutic strategy for treating AD [169]. Utilizing hydroxyquinoline and EGCG moieties to create the lead chemical TGR86, which may sequester Cu^2+^ from the A complex and enhance neuronal cell survival, is another novel therapeutic approach using metal chelators for treating AD. Flavonoids possess natural Fe and Cu chelating abilities. It was shown that Zn chelator (N, N, N′, N′-tetrakis (2-pyridinylmethyl)-1,2-ethylenediamine-TPEN) decreased Zn levels and increased longevity and health in transgenic C. elegans. Deferoxamine, deferasirox, and deferiprone as Fe chelators, trientine (TETA) as Cu chelators, and EDTA as a Zn chelator are further potential metal chelators that might be employed as possible treatment strategies [153].

## 10. Clinical Trials

Clinical trials using nanotheranostic approaches in AD represent a promising frontier in medical research, combining diagnostics and therapeutics into a single nanotechnology-based platform. These advanced systems aim to enhance the precision and efficacy of AD treatment by enabling early diagnosis and targeted drug delivery. Early trials have shown potential in improving cognitive function and slowing disease progression, offering hope for more effective management of AD through personalized and minimally invasive interventions. Table 2 demonstrates the only available trial using a nanotheranostic approach in AD.

## 11. Challenges Associated with Nanotheranostic Approaches

NPs are known to have widespread biological applications as drug delivery mechanisms due to their exceptional physicochemical and behavioral properties. However, there are significant uncertainties about the safety of manufactured NPs in humans as their use in biological applications expands. Because of their minute size and distinctive characteristics, NPs are frequently used in nanomedicine and as drug carriers [176]. However, the toxicity towards healthy human cells, tissues, and organs may also be owing to their crystallinity, solubility, aggregation, surface characteristics, morphology, surface area, and dose-dependent characteristics [177,178,179]. The ability of the NPs to trigger an innate immune response must be ascertained. Numerous metal and metal-oxide NPs have been reported to induce pro-inflammatory effects in both in vitro and in vivo studies [180]. NPs predominantly enter the human body through the respiratory system, wherein they frequently cause inflammatory reactions due to redox stress. Additionally, NPs can enter the brain. The olfactory nerve is hypothesized to carry NPs that impact the olfactory mucosa, from where they can travel to the brain and affect brain health and functioning [180]. Furthermore, oxidative stress is one of the most frequently studied effects of NPs. Increased production of ROS, which is preferred above antioxidants, leads to an oxidative stress condition. By-products of biological reactions, such as peroxynitrite (ONOO), nitric oxide (NO), hydroxyl radical (OH), hydrogen peroxide (H_2_O_2_), and superoxide radical (O_2_^−^), are the most prevalent ways of ROS generation [181]. The ROS damages proteins, lipids, and the most important biomolecules, which can activate a system similar to NADPH, disrupt the electron transport chain, depolarize the mitochondrial membrane, and affect the mitochondrial structure [182]. In a study by Hou et al., ZnO NPs (ZnONPs) cause DNA replication disorders in the cell cycle pathway’s G1, M, and G2 phases and the failure of mini-chromosome maintenance [183]. NPs also induce cytotoxicity by altering the numerous physicochemical, metabolic, and molecular pathways. Smaller NPs often have bigger surface areas, allowing interactions with cell constituents such as carbohydrates, fatty acids, proteins, and nucleic acids, suggesting that particle size may influence cytotoxic efficacy. A major contributor to cytotoxicity, associated with energy and metabolic abnormalities and cellular dysregulation, is the disturbance of Ca^2+^ (intracellular calcium). Even though Ca^2+^ is one of the key signaling molecules associated with signal transduction in metabolic regulations, its elevation has acute toxicity on cellular mitochondria, which results in the induction of apoptosis by preferentially releasing cytochrome c or by enhanced ROS production and opening the inner mitochondrial pore, eventually leading to the death of the individual [184]. The cytotoxic action of NPs has recently been demonstrated to cause cell death and suppress cell growth if cells are arrested in at least one cell cycle phase (G2/M phase, S phase, or G0/G1 phase). Cells arrested in the cell cycle either build up significant damage that causes apoptosis or repair the damage. Cell cycle arrest can be specific to certain cell types at particular stages. Regarding the study conducted by Gao et al., nickel oxide NP (NiONP) treatment resulted in a much lower G0/G1 phase in the A549 cell line and a significantly higher G0/G1 phase in the BEAS-2B cell line. This type of nanoparticle also impacts the cell cycle. ZnONP and CuONP exposure in T-cells resulted in G2/M phase arrest, while TiO_2_ resulted in S-phase arrest [185]. The primary cause of the process underlying NP-associated genotoxicity is the increased production of reactive nitrogen species (RNS) and ROS, which causes higher oxidative stress and damage to the genetic makeup. The interaction of the NPs with the DNA involves primary toxicity, whereas ROS/RNS contributes secondary genotoxicity to the NPs’ production. In the indirect primary clastogenic pathway, unsaturated aldehydes produced from primary lipid oxidation by ROS are used to produce exocyclic DNA adducts [182]. Numerous studies have documented the development of NPs like copper oxide (CuO) on biomedical platforms; nevertheless, they may have the potential to accelerate the process of protein oligomerization. A study by Jaragh-Alhadad and Falahati sought to comprehend how CuONPs affected the oligomerization of Aβ_1–42_ and related neurotoxicity. The study revealed crucial facts concerning the detrimental effects of CuONPs against CNS proteins that encourage the development of cytotoxic oligomers [186]. The analysis of nanoparticle toxicity will pave the way for developing better and more efficient NPs.

Off-target effects are a major challenge in drug delivery due to the barrier’s selective permeability and the presence of similar receptors in peripheral organs. To enhance the specificity of targeting brain endothelial cells, researchers have developed a sophisticated two-step targeting strategy.

### 11.1. Pre-Targeting with Biotinylated PECAM-1 Antibody

The first step involves using a biotinylated PECAM-1 antibody as a ligand. PECAM-1 (Platelet Endothelial Cell Adhesion Molecule-1) is a receptor found on the surface of endothelial cells throughout the body, including in both peripheral organs and the brain. The biotinylated PECAM-1 antibody binds to PECAM-1 receptors on endothelial cells. Peripheral endothelial cells, such as those in the lungs, have higher endocytic rates compared to brain endothelial cells. This means that, once the biotinylated antibody binds to the PECAM-1 receptor on these peripheral cells, the complex is quickly internalized and removed from the cell surface [187].

In contrast, brain endothelial cells, which line the BBB, have lower endocytic rates. As a result, the PECAM-1 antibody-receptor complex remains on the surface of these cells for a longer period, providing a window of opportunity for selective targeting [187].

### 11.2. Specific Binding with Avidin-Functionalized Nanoparticles

In the second step, polymeric nanoparticles functionalized with avidin are introduced. Avidin has a high affinity for biotin, so it binds specifically to the biotinylated PECAM-1 antibody that remains on the brain endothelial cell surfaces. This step ensures that the nanoparticles are directed primarily to the brain, where they can cross the BBB more effectively [187].

This two-step targeting strategy minimizes the accumulation of nanoparticles in peripheral organs, such as the lungs, heart, and pancreas, by taking advantage of the differential endocytic rates and specific binding interactions. The result is enhanced selective delivery of therapeutics to the brain, potentially improving treatment outcomes for neurological conditions while reducing off-target effects [187].

Extensive research is thus being carried out to increase the overall knowledge of NPs’ effects on the environment and public health and to advance the development of safer materials.

## 12. Future Scopes and Prospects

**Figure 6:**
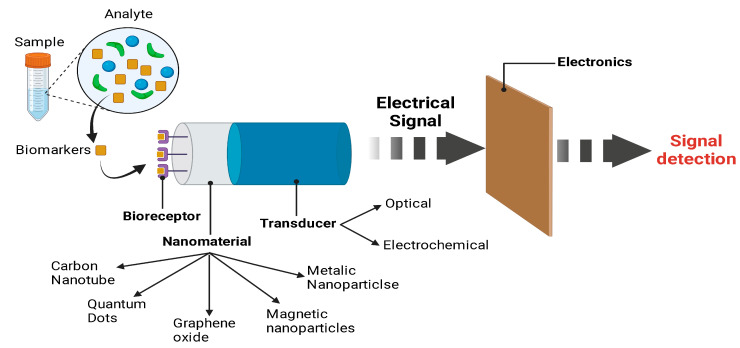
Schematic representation of a biosensor device for detecting biomarkers in a sample. The device consists of a bioreceptor component where specific biomarkers from the sample bind to the surface. Various nanomaterials such as carbon nanotubes, quantum dots, graphene oxide, metallic nanoparticles, and magnetic nanoparticles are used to enhance the specificity and sensitivity of the bioreceptor. In contact with the bioreceptor, the transducer element converts the biochemical signal into an electrical signal through either optical or electrochemical means. This signal is then relayed to the electronics component, which processes the signal for subsequent detection and quantification of the analyte (created with BioRender.com).

It has been stated by the World Health Organization (WHO), based on various estimations, that the frequency of individuals with dementia will massively quadruple in the coming decade, with almost 131 million people worldwide by 2050 [103]. Unsatisfactory outcomes from the clinical trials of AD treatments have highlighted the need for the further standardization of target populations and monitoring techniques. The BBB, which prevents conventional medications from entering the CNS, is another impenetrable barrier to treating AD. The most challenging topics in modern medicine may be methods for early diagnosis and treatment actions. The symptoms of AD can be minimized and temporarily slowed down by the currently available drugs, but the progression of brain damage cannot be prevented [188]. Significant progress has been made in the field of nanotechnology over the past years, particularly in the fields of medicine and material science. The medical use of nanotechnologies has greatly influenced the production of different drug-loaded nanocarriers, typically ranging in size from 1 to 1000 nm [188]. As an alternative to traditional medication delivery techniques, nanotechnology thus offers new possibilities for treating AD. According to recent research, NPs can successfully penetrate the BBB and exhibit suppressive action to increase specificity and efficiency at optimum pH and temperatures. Additionally, mitochondria-targeted therapeutic AD therapies may be expanded beyond in vitro and in vivo studies to human clinical trials. The nanocarrier’s characteristics, morphology, efficacy, and targeted delivery effectiveness can be enhanced by applying surfactants or hydrophilic substances like PEG to the surface of NPs, thus enhancing the therapeutic efficacy of AD [98,189]. Another popular area of research involves using stem cell therapeutics and nanotechnology to treat AD. This new technique controls stem cell proliferation and differentiation or uses nanotechnology to stimulate tissue healing and repair [190]. Artificial Intelligence (AI) may provide a broad spectrum of approaches for assessing massive and complicated data for a profound understanding of AD. It also emphasizes using computer-assisted diagnostic equipment for diagnosing AD and the prospective application of AI to clinical procedures to predict individuals at risk and patient categorization to eventually design streamlined and customized remedies [179]. With the use of promising analytical techniques of bioinformatics and statistics on Big Data in AD research projects, it is feasible to associate authorized subject matter expertise from psychology, neuroscience, neurobiology, psychiatry, geriatric medicine, biology, and genetics, thus aiming to provide comprehensive answers through the application of predictive prototypes [191]. It also provides significant insights into the ailment pathogenesis, patient categories, and a blend of suitable biomarkers, paving the way for establishing impactful treatment regimens and personalized patient medications [192]. Extensive research has been conducted to enhance our understanding of complex, multifaceted diseases like AD. AI capitalizes on machine learning and deep learning to construct algorithms that can be applied in biomedical and clinical setups to automate, standardize, and enhance the precision of early diagnosis and patient categorization according to the computation of relevant data [193]. AI thus holds great potential as a technique for advancing research and, ideally, providing novel personalized therapeutics [191]. Among the most effective biomedical breakthroughs for preventive measures against the disease are vaccines. The fundamental reason that vaccines are designed is to induce a preventive response without the constant necessity of administering a therapeutic drug via passive immunity [194]. Several peptide vaccine approaches have advanced to clinical trials, demonstrating that specific immune reactions may eliminate aggregates from the brain or halt their formation in the first place [195]. Different N-terminal segments of Aβ are used in several vaccine prototypes. The immunostimulatory characteristic of the Aβ peptide plays a role in targeting the N-terminus, which comprises B-cell epitopes in contrast to the C-terminus, which possesses T-cell epitopes. Formulating an Aβ vaccine that causes a strong anti-Aβ B-cell action without activating Aβ-specific T cells is therefore of high relevance. Targeting the correct pathological oligomeric form of Aβ or tau in next-generation immune therapeutics for AD may be crucial [196]. However, the fact that immunogenic reactions and responses are still exceedingly challenging to predict makes peptide vaccines a persistent concern [197]. Furthermore, ligand targets provide a personalized liposome that can speed up the rate drugs accumulate in the intended tissue. The liposome enables better monitoring of how long the therapeutic substance remains in the bloodstream, decreasing toxicity and extending the therapeutic activity [151]. Despite numerous research studies, there is still much work to be undertaken to apply nanotechnology in AD treatment (Figure 6). More powerful and non-toxic nanomedicine formulations are essential for effective medication delivery to treat patients with CNS illnesses like AD [198]. In addition, many other related works might be interesting to biotechnology and biomedical researchers [145,146,147,198].

## 13. Conclusions

Public health initiatives are focused on preventing AD globally. The intricacy of the disease’s symptoms and etiology, our inadequate understanding of its mechanism, and the possibility of a dormant, asymptomatic preclinical period all contribute to the challenge of treating AD. The usage of tailored medications is straightforward, despite numerous drugs continually being evaluated in clinical research for the treatment of AD due to the unique lack of patient response and occasionally severe adverse effects. Nanotheranostic approaches, or the application of nanotechnology, is one of the most critical developments in treating AD and other related disorders. Nanotechnology has the potential to revolutionize the treatment of neurodegenerative diseases by inducing biological responses at target sites while reducing side effects. The BBB shields the brain from toxic drugs, making drug delivery across the BBB difficult for neurodegenerative disease detection, localization, and therapy. Traditional medications frequently fail to cross the BBB, making them ineffective for treating the disease. Even though nanotechnology is being experimented with vastly, translational relevance and safety concerns are to be analyzed. This necessitates a thorough knowledge of how body systems interact with nanomaterials. It has been widely documented that QDs, metal NPs, and nanocomposites can cure various neurodegenerative diseases. It is necessary to overcome the restrictions put on these NPs. Although the research that has been conducted in the field has not produced any noteworthy outcomes that could be applied to humans, their effects on the lowering of molecular events leading to neurodegenerative disorders have been notable, so the strategic approach to popularize the use of nanotherapies instead of conventional drugs has a good chance of producing prominent outcomes shortly.

## Figures and Tables

**Table 1:** Potential nanotheranostic approaches for AD.

Specific Nanosystem Name	Type	Strategy	Size	Conjugated Drugs	Target	Main Function	References
Carbon Nanotubes	Inorganic NPs	In vivo/in vitro	1–100 nm	Berberine	BBB transcytosis, Cholinergic systems	Reduces Aβ accumulation	[98]
Lipid carriers	Organic NPs	Ex vivo	211.4 ± 3.54 nm	Curcumin	Amyloid cascades	Reduces Aβ burden	[99]
		In vivo/in vitro		Curcumin and nerve growth	BBB transcytosis, Amyloid cascades and tau hyperphosphorylation	Reduces Aβ plaque deposition and lowers AChE activity inside the hippocampus of AD rats	[99]
Polymeric NPs	Organic NPs	In vivo/in vitro	161.3 ± 4.7 nm	RVG29 peptide and tau tangles BACE1- AS shRNA gene As	Amyloid cascades	Primarily suppresses Aβ plaque burden and reduces phosphorylated-tau-tangles formation	[100]
Metal NPs	Inorganic NPs		1–100 nm	Anthocyanin	Amyloid cascades and tau Hyperphosphorylation	Anthocyanin-loaded PEG-AuNPs can exhibit neuroprotective potential in comparison to their free form by regulating the p-PI3K/p-Akt/p-GSK3b pathway, inhibition of tau hyperphosphorylation and amyloid cascades formation in AD mice model	[98]
				CLPFFD peptide	Amyloid cascades	PEGlyation of AuNP_S_, effective stabilization of the NPs through masking its negative charge and by facilitating BBB transport improved functionalization with CLPFFD peptide and enhancement of their selective binding toward amyloid fibrils	[98]
Magnetic NPs	Inorganic NPs	In vitro	<70 nm	Anti-transferrin monoclonal antibody (OX-26)	Amyloid cascades	Hindrance formation of extracellular accretion of Aβ aggregates	[101]
				Iron oxide	Amyloid cascades	The larger the concentration of NPs, the more will fibrillation in a magnetic field, whereas a smaller concentration downregulates it Surprisingly, negatively charged or uncharged nanoparticles show better fibrillation suppression	[98]
Quantum dots	Inorganic NPs	In vitro	1–100 nm	QD-biphenyl ethers	Amyloid cascades	Aβ fibril formation inhibition	[98]
		In vitro		Graphene quantum dots	Amyloid cascades	Aβ aggregation inhibition	[102]
Liposomal			10–100 nm	Curcumin derivative	Aβ, Cholinergic dysfunction	Represented as a carrier molecule	[103]
				Peptide Aβ	BBB transcytosis, Amyloid cascades		[103]
AuNPs			1–150 nm	Anthocyanin	Amyloid cascades and tau hyperphosphorylation	Affects different biological activities related to AD	[104]
				CLPFFD peptide	Amyloid cascades		[103]
Mesoporous silica NPs (MSN)			2–50 nm	Rivastigmine hydrogen tartrate	Neuronal cell death/Cholinergic systems	Acts as carrier molecules	[105]
				Metal chelator 5-chloro-4-hydroxy-7-iodoquinoline	BBB transcytosis, Amyloid cascades		[103]
Carbon dots			1–10 nm	Tunable zero-dimension	Acetylcholinesterase enzyme, Amyloid cascades	Take part in theranostic	[103]
Dendrimers	Organic NPs		1–10 nm	o-phenylene diamine	Amyloid cascades	Functions as carrier molecules and different chemical loading abilities can be carried to different brain parts	[106]
Nanoliposome (1,2-distearoyl-sn-glycero-3-phosphocholine; cholesterol)			110 ± 6 nm	Curcumin		Aβ fibril formation retardation	[103]
Liposome (Shirasu porous glass + cholesterol)			102 ± 2 nm	Modulate tau phosphorylation and glycogen synthase kinase 3 activities		Reduced Aβ clearance	[107]
Retro-Inverso peptide inhibitor nanoparticles			131 ± 43 nm	Inhibitors of aggregation of the Alzheimer’s Aβ peptide		Reduced Aβ clearance	[103]
Iron oxide			250–350 nm	Inhibitors of aggregation of the Alzheimer’s Aβ peptide		Inhibited formation of Aβ oligomers and fibrils in vitro	[103]
				H_2_O_2_-responsive therapy		Interfered with Aβ aggregation and neurotoxicity	
				Z-DEVD-FMK (caspase-3 inhibitor)		Decreased infarct volume, neurological deficit, and caspase-3 activity	
Chitosan			650 ± 2 nm	Z-DEVD-FMK and bFGF		Low infarct volume; improved motor function	[108]
Cationic Bovine Serum Albumin			114 ± 14 nm	Tanshinone IIA		Low infarct volume, neurological function deficit, neutrophil infiltration, and ultimately neuronal apoptosis	[103]
Lipidic (Squalene)			120 nm	Adenosine		Lower infarct volume; improved neurological deficit scores	[109]
Polylactic acid			118.3 ± 7.8 nm	nanoparticles angiopep-2-conjugated, 125 NAP (NAPVSIPQ)-loaded (NAP: neuroprotective peptide)		Increased drug uptake inside the brain, impairment in ameliorated learning, cholinergic disruption, and functional loss of hippocampal neurons	[103]
Poly(butylcyanoacrylate)			250 ± 30 nm	Nerve growth factor		Reversed scopolamine-induced amnesia and improvement in recognition and memory	[108]
Carboxyl-conjugated AuNPs (negatively charged)			250 ± 30 nm	Negatively charged AuNPs		Disrupted the Aβ fibrillation and fragmented the fibrils already that were formed	[103]

This table completely enumerates the potential nanotheranostic approaches and their respective size, types, strategies, conjugated drugs, and primary functions. NPs: nanoparticles, AuNPs: gold nanoparticles. Any other abbreviations must be described.

**Table 2:** Currently available nanotheranostic-based clinical trial for AD therapy.

Trial ID	Intervention	Clinical Importance
NCT03806478	Intranasal Nanoparticles of APH-1105	Phase 2 study assessing the safety, tolerability, and efficacy of intranasal delivery of APH-1105 for treating mild to moderate AD in adults.
